# Potential threats of environmental microplastics to the skeletal system: current insights and future directions

**DOI:** 10.3389/fendo.2025.1658056

**Published:** 2025-09-09

**Authors:** Haofan Zhao, Sirong Mu, Weizhou Wang, Xi Li

**Affiliations:** ^1^ Department of Orthopedics, The First Affiliated Hospital of Kunming Medical University, Kunming, Yunnan, China; ^2^ The First Clinical Medical College, The First Affiliated Hospital of Kunming Medical University, Kunming, Yunnan, China

**Keywords:** microplastics, nanoplastics, bone microenvironment, osteoblast, osteoclast, environmental toxicology

## Abstract

Micro- and nanoplastics (MNPs), as emerging environmental pollutants, have attracted global attention due to their pervasive presence in ecosystems and human living environments. Plastic additives confer high durability, and MNPs derived from environmental degradation can enter the human body via inhalation or ingestion. Smaller particles are capable of penetrating biological barriers and accumulating in various tissues. Recent studies have revealed that beyond their known accumulation in the digestive, respiratory, and reproductive systems, MNPs may also reach the bone microenvironment via systemic circulation. The bone microenvironment, composed of diverse cell types and extracellular matrix components, is essential for maintaining bone formation, remodeling, and immune regulation. Emerging evidence indicates that MNPs can infiltrate the bone marrow, disrupt its homeostasis, and accumulate within the musculoskeletal system, potentially impairing bone metabolism and function. This review summarizes the sources and metabolic pathways of MNPs and elucidates their impact on the bone microenvironment, with a focus on mechanisms involving chemical toxicity, inflammation, and metabolic dysregulation. The findings provide a theoretical foundation and research direction for evaluating the risks of MNPs exposure to skeletal health.

## 1 Introduction

In recent years, global plastic production has exceeded 400 million tons annually, yet since 2012, the overall recycling rate has remained as low as 9% (1, 2). A significant portion of plastic waste ultimately enters the natural environment. For instance, in 2019, Europe generated approximately 25.9 million tons of plastic waste, the vast majority of which was inadequately treated (3). Over time, environmental weathering leads to the breakdown of plastics into microplastics (MPs, 1 µm–5 mm) and nanoplastics (NPs, <1 µm), collectively referred to as MNPs. Studies have shown that MNPs can enter the human body through various exposure routes, including dietary intake, drinking water, and airborne particles. It is estimated that the average person ingests approximately 0.1–5.0 grams of MNPs per week, equating to 74,000–121,000 particles annually (4, 5). Due to their widespread presence and potential toxicity in both ecosystems and the human body, MNPs have emerged as a pressing global public health concern (6). Accumulating evidence suggests that MNPs can activate multiple pathophysiological pathways associated with skeletal toxicity, affecting the function of chondrocytes, osteoblasts, and osteoclasts (7). These effects are often mediated through oxidative stress, inflammatory responses, and disruptions to bone homeostasis (8). Animal studies further support that MNPs exposure can lead to structural and metabolic abnormalities in bone tissue (9). To ensure a comprehensive and systematic synthesis of existing evidence, we performed database searches in PubMed, Web of Science, and Scopus from inception to June 2025. The primary search strategy was: (“microplastics” OR “nanoplastics”) AND (“osteotoxicity” OR “bone” OR “skeletal system” OR “osteoblast” OR “osteoclast” OR “bone microenvironment” OR “bone remodeling”). Only peer-reviewed articles published in English were considered. Inclusion criteria encompassed (i) original studies using *in vivo* mammalian models or *in vitro* mammalian cells, (ii) investigations directly assessing the effects of microplastics or nanoplastics on bone, bone marrow, or the skeletal muscle system, and (iii) availability of full text. Exclusion criteria were: (i) editorials, commentaries, conference abstracts, and case reports, (ii) studies not directly related to musculoskeletal outcomes, (iii) non-English publications, and (iv) studies lacking sufficient methodological detail or quantitative data relevant to the musculoskeletal system. The primary aim of this review is to synthesize the current scientific evidence on the effects of MNPs on the musculoskeletal system, with an emphasis on the emerging research landscape. It focuses on studies reporting the detection of MNPs within the musculoskeletal system and explores their potential associations with skeletal disorders. Finally, this review outlines future research directions necessary to elucidate the scientific basis of MNPs as an emerging risk factor for bone health.

## 2 What are microplastics? Routes of human exposure and metabolic fate

Since the invention of plastic by Belgian chemist Leo Baekeland in 1909, the variety and applications of plastic materials have expanded rapidly. To meet diverse functional requirements, plastic products are commonly formulated with additives such as antioxidants, plasticizers, and flame retardants (10), which enhance their durability and render them persistent organic pollutants. Through biotic and abiotic degradation processes in natural environments and biological systems, plastics can break down into MNPs, classified by particle size into microplastics (MPs,1 µm–5 mm) and nanoplastics (NPs, <1 µm) (11). MNPs can be either primary—manufactured at microscopic sizes—or secondary, generated from the breakdown of larger plastic debris. They are widely found in personal care products, synthetic materials, and fragmented plastic waste (12). Common polymers such as polyethylene (PE), polypropylene (PP), polystyrene (PS), polyvinyl chloride (PVC), and polyethylene terephthalate (PET) account for approximately 90% of global plastic production (13). These polymers differ significantly in their environmental degradability, making complete elimination of plastic pollution particularly challenging. Recent studies have demonstrated that MNPs can be taken up by plants and transferred through the food chain to animals and humans, raising global concerns regarding their potential toxicity and health risks (14).

Given their near-ubiquitous presence across global ecosystems, MNPs have emerged as a growing environmental threat (15). Facilitated by wind, riverine systems, and ocean currents, MNPs can undergo long-range transport and are now distributed across aquatic systems, terrestrial soils, and the atmosphere (16). Research has shown that MNPs can enter organisms via inhalation, ingestion, or foliar absorption, potentially impairing reproductive and physiological health, and ultimately bioaccumulating in the human body through trophic transfer (14, 17, 18). Although larger particles are often excreted via feces, smaller MNPs are more readily absorbed and biologically active (19). Particles smaller than 150 μm can penetrate intestinal epithelial cells, while those under 20 μm may translocate via M cells or dendritic cells to distant organs such as the liver, kidneys, and muscles (20). Particles ranging from 0.1 to 10 μm in size have even been shown to cross the blood–brain barrier (21). Moreover, MPs can further degrade into nanoplastics capable of integrating into cellular membranes (22), and their accumulation in various human tissues has now been confirmed (23). Animal studies have also demonstrated that MNPs can enter the body via ingestion, inhalation, or dermal contact, triggering a range of health risks (24). Despite increasing evidence of MNP accumulation and toxicity in multiple human tissues, their behavior and effects within bone tissue remain poorly characterized. Given the structural complexity of the bone microenvironment and its essential roles in bone metabolism and immune regulation, elucidating how MNPs enter and impact this niche is critical to advancing our understanding of their biosafety and systemic health implications.

**Figure 1:**
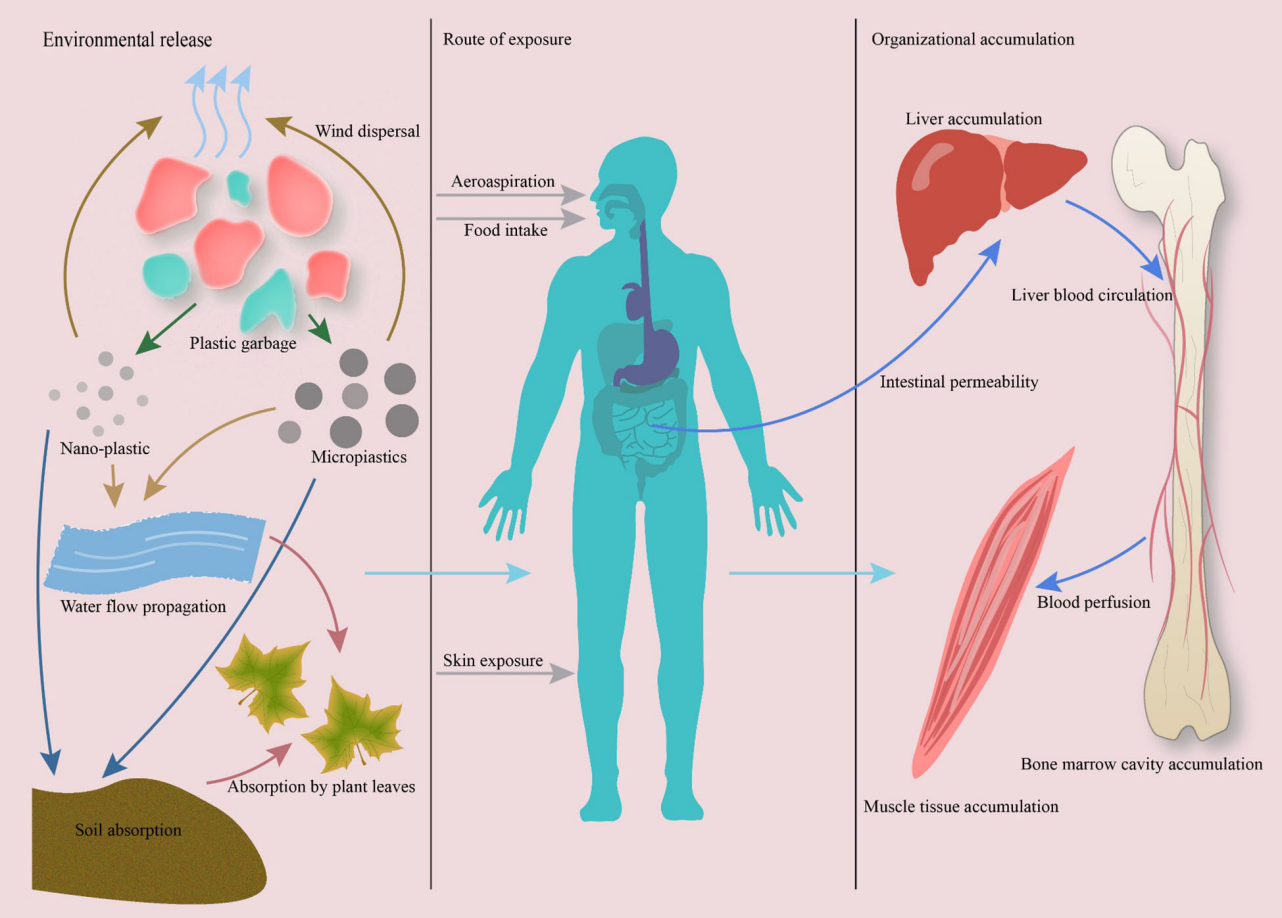
Pathways of micro- and nanoplastics (MNPs) exposure and accumulation in the human body. MNPs enter ecosystems via plastic degradation and are ingested or inhaled by humans. Smaller particles (<20 μm) penetrate biological barriers and disseminate through systemic circulation, accumulating in bone marrow and musculoskeletal tissues. Evidence from human and animal studies confirms MNP presence in bone, highlighting their potential to disrupt skeletal homeostasis.

Although MPs and NPs often share similar environmental sources and exposure routes, their particle size–dependent physicochemical properties lead to notable differences in their *in vivo* distribution, biological effects, and toxicological characteristics. In terms of distribution, MPs—particularly those larger than 150 µm—are generally retained within the gastrointestinal tract or translocated to specific organs through lymphatic and circulatory pathways, whereas NPs can penetrate cellular membranes more readily, enter systemic circulation, and cross critical biological barriers such as the blood–brain barrier and placenta. Regarding biological effects, MPs tend to induce localized tissue irritation, chronic inflammation, and microbiome dysbiosis, while NPs, due to their higher surface area-to-volume ratio and reactivity, can directly interact with intracellular components, disrupt mitochondrial function, and alter organelle dynamics. In terms of toxicological characteristics, NPs generally exhibit higher bioavailability, stronger potential for biomolecular binding, and greater potency in inducing oxidative stress and apoptosis at lower doses compared with MPs. Nevertheless, both MPs and NPs are capable of perturbing bone homeostasis through mechanisms involving oxidative stress, inflammatory signaling, and dysregulation of key pathways such as NF-κB, MAPK, and BMP/Smad, ultimately leading to altered bone remodeling and skeletal integrity.” As summarized in **Figure 1**, MNPs can enter the human body via ingestion, inhalation, or dermal absorption, and smaller particles penetrate biological barriers to reach distant tissues including bone marrow.”

## 3 Effects of MNPs on the bone microenvironment

The bone microenvironment refers to the intricate biological niche surrounding bone tissue, composed of osteoblasts, osteoclasts, bone marrow mesenchymal stem cells, as well as extracellular matrix components, vascular networks, cytokines, and signaling pathways (25). This specialized milieu not only supports bone formation, remodeling, and repair, but also plays crucial roles in immune regulation, hematopoiesis, and tumor metastasis (26). Emerging evidence indicates that MNPs can penetrate biological membranes and interact with cells (27).To date, MNPs have been detected in human colon, testis, endometrium, placenta, and lung tissues (28–32), suggesting that they can enter the body via inhalation, ingestion, or the circulatory system, accumulate in various organs, and exert potential toxicity on the reproductive, nervous, and digestive systems (33). These findings also imply that MNPs may target the bone microenvironment. Studies have identified nanoplastic particles composed of PET, PS, or PE in human peripheral blood, with an average concentration of 1.6 μg/mL (34). Moreover, bone marrow samples have been shown to contain PE, PS, polyvinyl chloride (PVC), polyamide-66 (PA66), and PP, with an average concentration of 51.29 μg/g (35). Given that bone tissue receives nutrients primarily through the dense vascular networks of the periosteum, subchondral bone, and endplate regions (36, 37) there exists a plausible anatomical basis for MNPS entry into the bone microenvironment. More importantly, polystyrene nanoparticles with diameters less than 100 nm have been shown to infiltrate the bone marrow, impair hematopoietic function, and disrupt the homeostasis of the bone marrow niche (35, 38). Recent human studies also suggest that MNPs may accumulate within the musculoskeletal system and contribute to various pathological processes (39). Collectively, these findings indicate that MNPs can access bone tissue via the circulatory system and may disturb the bone microenvironment through mechanisms including physical damage, chemical toxicity, inflammatory activation, and metabolic disruption, posing a potential threat to bone homeostasis and function.

### 3.1 Effects of MNPs on bone resorption

Osteoclasts are multinucleated, macrophage-like cells derived from the monocyte–macrophage lineage. Their primary function is to mediate bone resorption and remodeling, playing essential roles in skeletal development, bone homeostasis, and maintenance of the hematopoietic microenvironment (40). The differentiation of osteoclasts is regulated by a variety of factors; however, the epigenetic mechanisms determining the fate of monocyte–macrophage progenitors during differentiation into human osteoclasts remain incompletely understood. It is well established that the key factor regulating osteoclastogenesis is receptor activator of nuclear factor κB ligand (RANKL), a member of the tumor necrosis factor (TNF) family, expressed on macrophage precursor cells, and regarded as a principal osteoclastogenic cytokine (41, 42). By binding to its receptor Receptor activator of nuclear factor κB (RANK), RANKL activates multiple transcriptional pathways, including Nuclear factor κB (NF-κB), CEBPα,AP-1(c-Fos and c-Jun),and MAPK family members (ERK, JNK, and p38), which together drive the expression of the master transcription factor Nuclear factor of activated T-cells, cytoplasmic 1 (NFATc1) (43–45). NFATc1 subsequently translocates into the nucleus and, in cooperation with other transcription factors, orchestrates osteoclast differentiation (46), ultimately giving rise to mature, multinucleated bone-resorbing cells.

In recent years, increasing attention has been directed toward the disruptive effects of MNPs on osteoclast differentiation and function. An *in vitro* study demonstrated that MNPs could induce oxidative stress and activate the p38 and JNK MAPK signaling pathways through the phosphorylation of JNK and p38 MAPKs, ultimately resulting in an increased number of osteoclasts (47). In another study, Pan et al. (48) established a chronic low-dose exposure model to polystyrene microplastics (PS-MPs) to investigate their impact on the skeletal system. The results revealed substantial deterioration of femoral microarchitecture in exposed mice, characterized by a marked reduction in trabecular number and significant bone loss, indicating a disruption of bone homeostasis. The authors further demonstrated that PS-MPs activated the NF-κB signaling pathway to enhance osteoclast activation and bone resorption, while interfering with osteoclast differentiation. Notably, administration of an NF-κB inhibitor *in vivo* reversed PS-MP–induced RANKL secretion and significantly reduced osteoclast formation.

Subsequent studies have shown that MNPs affect various bone cell types, with the most pronounced effects observed in RAW264.7 pre-osteoclasts, where exposure promoted their differentiation into mature osteoclasts (9). MNPs exposure led to the significant upregulation of osteoclastic catabolic markers, including genes such as TRAP, Npy, and Clc-7. TRAP is a classic marker of osteoclasts that enhances bone resorption activity (49); Npy, a multifunctional neuropeptide, not only promotes adipogenesis and inhibits osteogenic differentiation of mesenchymal stem cells but is also upregulated during aging and osteoporosis, while Npy deficiency in bone cells is associated with a high bone mass phenotype (50, 51); Clc-7 is a critical chloride channel located in the ruffled border of osteoclasts, essential for bone resorption (52). The upregulation of these genes suggests that MNPs exposure within the bone microenvironment not only increases osteoclast number but may also accelerate bone cell senescence, thereby exacerbating the imbalance between bone formation and resorption. In summary, these findings highlight the potential of MNPs to disrupt bone homeostasis by promoting osteoclastogenesis and impairing skeletal integrity.

To put it simply, MNPs can activate osteoclast differentiation by modulating key signaling pathways such as NF-κB and MAPK, and upregulate osteoclast-associated genes including TRAP, Npy, and Clc-7, thereby enhancing bone resorptive activity and disturbing the dynamic balance between bone formation and resorption, ultimately impairing skeletal homeostasis. Furthermore, the specific targeting of pre-osteoclasts by MNPs highlights their toxicological relevance in bone metabolism, suggesting that the NF-κB/MAPK axis may serve as a potential therapeutic target for mitigating MNP-induced skeletal toxicity.

### 3.2 Effects of MNPs on osteogenesis

Osteoblasts are key cells responsible for constructing bone structure, characterized by their pluripotency and critical functional roles. Their differentiation involves multiple stages and is tightly regulated at the molecular level to ensure proper skeletal development and maintenance of the bone microenvironment. Osteoblasts originate from mesenchymal stem cells (MSCs), which sequentially differentiate into chondro-osteoprogenitors, osteoprogenitors, and pre-osteoblasts, ultimately maturing into functional osteoblasts under the control of osteogenic transcription factors such as Runt-related transcription factor 2 (Runx2)and Osterix (Osx) (53, 54). This multistep differentiation process is regulated by several classical signaling pathways and transcription factors, including SRY-box transcription factor 9 (SOX9) (55), Runx2 (56), BMP2/BMP4 (57), and Wnt/β-catenin (58), all of which may be disrupted by MNPs exposure.

An *in vitro* study using zebrafish larvae revealed that exposure to polystyrene nanoplastics (PS-NPs) at 100 μg/mL for four days significantly increased the transcription levels of sp7, sparc, and smad1, while simultaneously downregulating runx2, bmp2b, and bmp4. These gene expression abnormalities were further exacerbated after seven days of exposure (8). The study suggested that PS-NPs exert skeletal toxicity by interfering with the BMP-Smad signaling pathway, ultimately disrupting normal bone development in zebrafish. Supporting this, another study reported that MP exposure significantly suppressed the expression of the osteogenic marker osteocalcin (OCN), as well as the transcriptional activity of the key regulator Runx2 (59). In addition, PS-MPs–treated femoral osteoblasts displayed clear signs of cellular senescence during late puberty. *In vivo* experiments further confirmed a reduction in both the number and length of trabeculae in mice, accompanied by a decline in OCN levels, suggesting a reduction in osteoblast numbers. Osteoblast senescence is considered a critical factor contributing to increased bone fragility, bone loss, and osteoporotic fractures (60, 61).

A separate study (9) assessed the migratory capacity of MC3T3-E1 cells exposed to MPs at concentrations ranging from 1 to 200 µg/mL, and conducted transcriptomic profiling post-exposure. The authors reported multiple alterations induced by MPs exposure, including increased reactive oxygen species (ROS) generation, activation of caspase-mediated apoptosis, and downregulation of key osteogenic genes such as OPG (osteoprotegerin) and IGF1 (insulin-like growth factor 1). Other studies have similarly shown that MNPs exposure can lead to a reduced RANKL/OPG ratio, thereby impairing endochondral ossification and reducing bone mass in juvenile rats (62). The RANKL/RANK/OPG signaling axis is one of the key regulators of bone remodeling (63), with the RANKL/OPG ratio playing a crucial role in osteogenic activity. OPG binds to RANKL and inhibits its interaction with RANK, thereby suppressing osteoclastogenesis. IGF1, a pivotal mediator of osteoblastogenesis and inflammation-related pathways, is also associated with the osteoclastogenic potential of pre-osteoclasts. Downregulation of IGF1 and OPG further exacerbates the imbalance between bone formation and resorption.

Meanwhile, upregulation of SOX9 expression may suppress chondrocyte hypertrophy and matrix remodeling, leading to disrupted processes of proliferation, differentiation, and apoptosis. Moreover, PS-MPs have been shown to induce senescence in bone marrow-derived mesenchymal stem cells (BMSCs) and disrupt their lineage commitment by activating the NF-κB signaling pathway (48). Senescent BMSCs exhibit a preferential differentiation toward adipocytes rather than osteoblasts, further undermining bone metabolic homeostasis (64). Similarly, another study employed a human BMMSC model to evaluate differentiation outcomes following exposure to 10 µg/mL of PET (65). The results demonstrated that MPs exposure significantly disrupted the fate determination of BMMSCs, partially eroding their stemness and inducing cellular senescence. Further analysis revealed a notable decline in osteogenic differentiation potential, accompanied by enhanced adipogenic commitment, evidenced by elevated expression of PPARγ (peroxisome proliferator-activated receptor gamma) mRNA. These findings are in line with earlier reports (48, 64).

**Figure 2:**
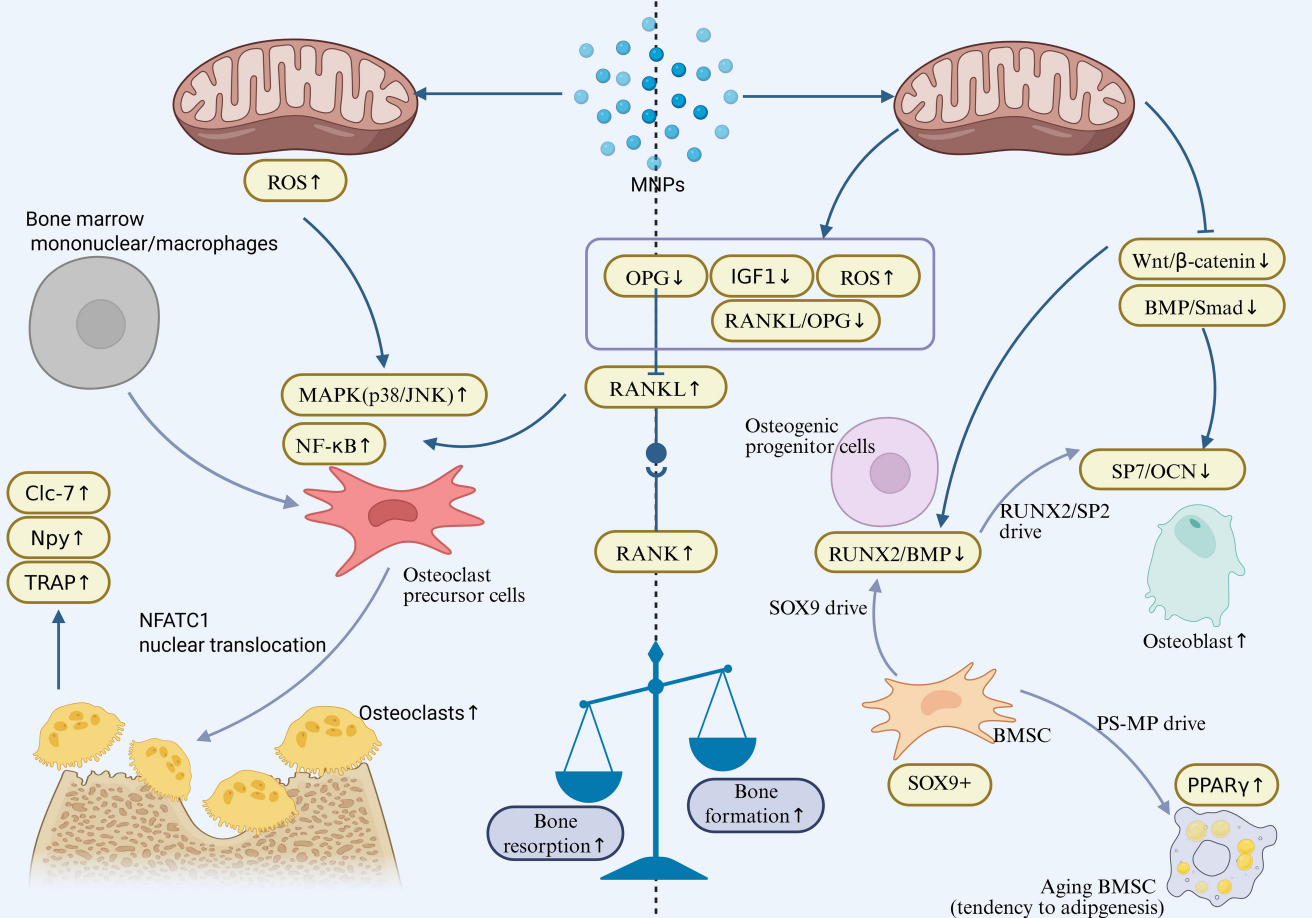
Osteoclast activation (Enhanced Bone Resorption):Activates the NF-κB/MAPK signaling axis (p38/JNK↑) in osteoclast precursors, inducing NFATc1 nuclear translocation and upregulating bone resorption genes (TRAP, Npy, CLC-7). Simultaneously increases the RANKL/OPG ratio (RANKL↑, OPG↓), promoting osteoclast differentiation and bone matrix degradation. Osteoblast Inhibition (Suppressed Bone Formation):Downregulates key osteogenic differentiation factors (Runx2/OCN) and the BMP/Smad pathway. Activates NF-κB-mediated senescence in BMSCs, enhances adipogenic propensity (PPARγ↑), and disrupts the RANKL/OPG balance (OPG↓, RANKL↑), ultimately leading to reduced bone formation. Final Outcome: Uncoupled bone resorption and formation results in bone loss and osteoporosis.

Taken together, these studies suggest that MNPs exposure can interfere with osteogenesis through multiple mechanisms. On one hand, MNPs inhibit osteoblast differentiation efficiency by downregulating SOX9 and disrupting key signaling pathways such as BMP/Smad/Runx2. On the other hand, MNPs induce premature senescence and apoptosis in osteoblasts, thereby impairing their bone-forming capacity, ultimately leading to the destruction of trabecular bone architecture and abnormal skeletal development. Furthermore, by disrupting stem cell fate determination and skewing differentiation away from the osteogenic lineage, MNPs further exacerbate the loss of osteogenic potential. Collectively, current evidence highlights the multifaceted and multi-targeted toxic effects of MNPs on bone tissue, emphasizing their potential risk in bone metabolic disorders and skeletal diseases. Thus, MNPs act on both arms of the remodeling process—enhancing bone resorption while diminishing bone formation. This combined disruption is captured in **Figure 2**, which illustrates the imbalance underlying MNP-induced skeletal toxicity.

### 3.3 Oxidative stress is a key factor in the impact of MNPs on the bone microenvironment

Bone remodeling is a dynamic process regulated by the coordinated actions of osteoclasts and osteoblasts (66). Under physiological conditions, a moderate level of ROS acts as signaling molecules that contribute to maintaining bone homeostasis (67, 68). However, excessive ROS can induce apoptosis of osteoblasts and osteocytes, inhibit bone formation, and eventually lead to reduced bone mass and osteoporosis (69, 70). Accumulating evidence indicates that ROS exert their disruptive effects on bone remodeling primarily through several key signaling pathways, including the RANKL/OPG axis, the Wnt/β-catenin pathway, and the NF-κB/MAPK pathways (69–71). Specifically, ROS upregulate RANKL expression while suppressing OPG, significantly increasing the RANKL/OPG ratio and thereby enhancing osteoclast activity (41). Meanwhile, ROS impair Wnt/β-catenin signaling, suppressing osteoblast differentiation (72, 73). In addition, ROS can activate the MAPK pathway, leading to inflammation and apoptosis, whereas nuclear factor erythroid 2-related factor 2 (Nrf2) serves as a critical antioxidant regulator involved in osteoclast regulation (74). Collectively, oxidative stress disturbs the balance of bone remodeling via multiple pathways, forming a fundamental mechanism underlying osteoporosis and other bone metabolic disorders.

Previous studies have demonstrated that exposure to PS-NPs at a concentration of 2.4 mg/mL can induce cellular senescence, promote adipogenic differentiation, and significantly modulate cell cycle progression in human bone marrow–derived mesenchymal stem cells (hBM-MSCs) (75). Mechanistically, such exposure enhanced the cells’ antioxidant capacity against reactive ROS, as evidenced by the upregulation of GPX3 (glutathione peroxidase 3) gene expression, while concurrently downregulating HSP-70 (heat shock protein 70) and XBP1 (X-box binding protein 1), both of which are closely associated with oxidative stress responses. Moreover, MNPs exposure markedly altered mitochondrial dynamics, with increased expression of the mitochondrial fusion-related gene Mitofusin-2 (MFN2)and decreased expression of the fission-related gene Mitochondrial fission 1 protein (FIS1), suggesting enhanced mitochondrial fusion activity. Cell cycle analysis further revealed an increased proportion of cells in the S phase following exposure, indicating elevated proliferative capacity, which may contribute to the observed adipogenic shift. Collectively, these findings suggest that MNPs exposure can profoundly influence the biological behavior of hBM-MSCs by modulating oxidative stress responses, mitochondrial function, and the expression of cell cycle–related genes.

MNPs have also emerged as critical exogenous disruptors of bone remodeling homeostasis. Numerous studies have confirmed that MNPs induce ROS production (8, 48, 62), and their impact on osteoblast function is closely associated with elevated ROS levels in the bone microenvironment. Excessive ROS suppress the activity and differentiation of osteoblasts, ultimately impairing bone formation and mineralization (76–78). Liu et al. (8) reported that exposure of zebrafish larvae to PS-NPs (10 and 100+ μg/mL) for 4 and 7 days resulted in a significant increase in ROS levels, along with decreased transcription of antioxidant enzyme genes sod1 and cat. Meanwhile, the expression of apoptosis-related genes bcl-2 and bax was upregulated, and persistent oxidative stress activated Caspase-3, triggering apoptosis in osteoblasts and leading to skeletal malformations. Nanoplastics also compromised membrane stability and mitochondrial function, increased ROS and nitrite production, and induced osteoblast necrosis and apoptosis—effects closely linked to ROS accumulation (79). Additionally, microplastic exposure can trigger endoplasmic reticulum (ER) stress in tibial chondrocytes, where excessive ROS lead to protein misfolding and activation of the unfolded protein response (UPR).This, in turn, suppresses protein synthesis, enhances chaperone and degradation system activity, and disrupts endochondral ossification within the growth plate (62).

*In vivo* and *in vitro* studies further demonstrate that MPs pose a systemic threat to bone health, involving physical stress, cell death, inflammation, and immune dysregulation (80). Exposure to MPs in animal models (3, 81, 82) and human cell lines (83) has been shown to induce excessive ROS production and oxidative stress (OS). ROS are highly reactive molecules involved in various biochemical processes. For instance, Jeong et al. (84) observed significantly elevated ROS levels in Daphnia magna following ingestion of 0.05 μm polystyrene microspheres, which activated the MAPK pathway and suppressed the Nrf2 pathway, further implicating ROS in microplastic-induced bone toxicity. Moreover, MPs accelerate osteoblast senescence, thereby hindering bone formation and contributing to degenerative skeletal changes (62). Literature also suggests that ROS are strongly implicated in skeletal aging (72), and that oxidative stress caused by increased ROS or impaired antioxidant defense systems is closely associated with osteoporosis (85). Elevated oxidative stress in cells leads to low bone turnover, and decreased bone mass is positively correlated with reduced antioxidant enzyme levels (86, 87). The disturbance of dynamic balance between osteoclasts and osteoblasts due to ROS accumulation ultimately results in decreased bone density and quality, weakening bone strength and increasing fracture risk—hallmarks of osteoporosis and age-related skeletal diseases (88).

**Figure 3:**
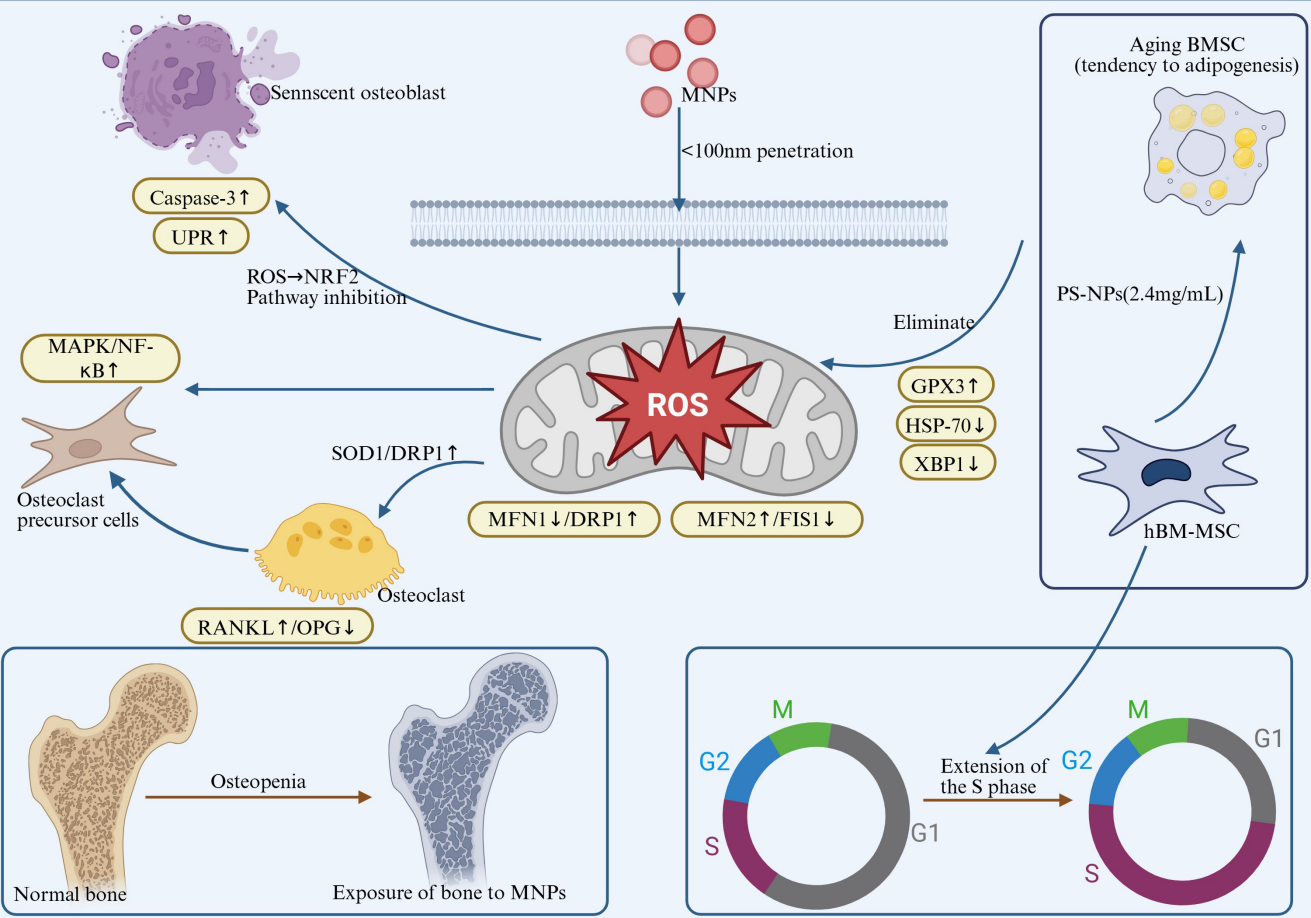
MNPs exposure induces ROS burst, triggering a tripartite toxic effect: a. Oxidative Damage: Inhibits antioxidant enzymes (SOD1/CAT↓) and activates caspase-3-mediated osteoblast apoptosis (Bax/Bcl-2↑). b. Organelle Dysfunction: Induces endoplasmic reticulum stress (UPR↑) and mitochondrial dynamic imbalance (MFN2↑/FIS1↓ → enhanced fusion). c. Metabolic Reprogramming: Upregulates GPX3 but inhibits HSP70/XBP1, prolongs the cell S phase, and promotes BMSC adipogenic differentiation. Pathological Outcomes: Osteocyte death, mineralization defects, and impaired bone remodeling, exacerbating the risk of osteoporosis.

In summary, MNPs promote excessive ROS generation, impair the functions of osteoblasts and osteoclasts, and disrupt the balance of bone remodeling, ultimately leading to reduced bone mass and osteoporosis. The underlying mechanisms involve activation of multiple signaling pathways, suppression of antioxidant systems, and induction of ER stress, collectively contributing to systemic bone toxicity. Future research should focus on elucidating the molecular mechanisms of MNPs-induced oxidative stress and exploring antioxidant-based intervention strategies, offering novel insights and therapeutic targets for bone-related diseases. As shown in **Figure 3**, excessive ROS induced by MNP exposure disrupts bone remodeling via multiple pathways, including RANKL/OPG imbalance, Wnt/β-catenin suppression, and MAPK activation.”

## 4 Effects of MNPs on skeletal muscle

The skeletal system and skeletal muscle are closely interconnected tissue systems that exhibit coordinated changes during development, growth, aging, and various pathological conditions. Skeletal muscle is not only one of the most abundant tissues in the human body—accounting for approximately 40–50% of total body weight in healthy adults—but also serves as a vital protein reservoir. In addition to regulating locomotor functions, skeletal muscle plays a fundamental role in respiration, feeding, energy metabolism, and the maintenance of glucose, amino acid, and lipid homeostasis, thereby being essential for overall health and quality of life (89).

Although studies have shown that MNPs can enter and accumulate in skeletal muscle tissue through dermal absorption or trophic transfer, current research on their impact on muscle structure and function is primarily limited to *in vitro* experiments and animal models, and the underlying mechanisms remain incompletely understood. For instance, Pause et al. (90)demonstrated that 100 nm polystyrene nanoparticles could penetrate granulosa cells in bovine oocytes and porcine myocytes, indicating their membrane-crossing capability. Similarly, Yang et al. (91) reported that feeding piglets with 150 mg/kg PS-MPs led to upregulation of THBS1 expression, which suppressed angiogenesis and subsequently impaired skeletal muscle development and meat quality traits such as flavor and redness. In chickens, Chen et al. (92) found that oral administration of PS-MPs for 21 consecutive days significantly increased microplastic accumulation in the pectoralis muscle, while levels decreased in the leg muscle over time. *In vitro* experiments further revealed that PS-MPs promoted both proliferation and apoptosis of primary chicken myoblasts, while inhibiting their differentiation, suggesting that MPs may modulate gene networks involved in neural function and muscle development, thereby affecting skeletal muscle physiology.

In addition, studies using fish models have demonstrated that co-exposure to microplastics and emamectin benzoate (EMB) induces oxidative stress and disrupts the balance of mitochondrial fusion and fission. Specifically, co-exposure led to the downregulation of fusion-related genes (Mfn1, Mfn2, OPA1) and upregulation of the fission-related gene DRP1, resulting in elevated levels of ROS, reduced mitochondrial membrane potential, and impaired ATP synthesis, ultimately contributing to skeletal muscle atrophy (93). Notably, the extent of damage under combined exposure was more severe than that induced by either agent alone, and supplementation with the antioxidant N-acetylcysteine (NAC) effectively alleviated these toxic effects. Consistent findings have been reported in C2C12 myoblasts, where PS-NP exposure was shown to induce mitochondrial dysfunction in differentiated muscle cells, disrupt cellular homeostasis, and promote a premature aging phenotype (94). Further evidence has demonstrated that maternal exposure to PS-NPs (0.1 μm, 10 mg/L) via drinking water during pregnancy altered the expression of genes related to lipid metabolism and muscle development in fetal muscle tissue and even interfered with skin formation (95). In another study, PS-MPs of different particle sizes (1–10 μm and 50–100 μm) were directly injected into the tibialis anterior muscle of mice. While no significant effect on overall muscle growth was observed, smaller particles markedly impeded fibrotic tissue repair following muscle injury, suggesting that particle size is a critical determinant of microplastic toxicity (96).

MPs can infiltrate the skeletal muscle system via various exposure routes and accumulate in muscle tissues across different species, though their distribution appears to be tissue-specific. MPs disrupt muscle cell homeostasis through mechanisms including oxidative stress, mitochondrial dysfunction, and gene dysregulation, leading to imbalances in myoblast proliferation and differentiation, impaired muscle fiber repair, and even atrophy. Notably, MP-induced toxicity is both size-dependent and potentiated under co-exposure conditions. However, most existing studies are limited to *in vitro* systems or agricultural species. Future research should focus on elucidating the long-term and transgenerational effects of MPs on human skeletal muscle and exploring the mechanistic differences induced by MPs with varying physicochemical properties, thereby providing a theoretical basis for strategies aimed at preserving muscle health.

To provide a comprehensive overview, **Table 1** consolidates the current evidence on how MNPs affect bone resorption, osteogenesis, oxidative stress, the bone marrow microenvironment, and skeletal muscle.”

**Table 1:** Effects of micro- and nanoplastics (MNPs) on the skeletal system.

Affected component	MNPs effects	Key molecular mechanisms/markers	Pathological outcomes	References
Bone resorption	Increased osteoclast differentiation and activity	NF-κB activation↑; MAPK (p38/JNK) activation↑	Bone resorption↑; Trabecular bone number↓	(47, 48)
		TRAP, Npy, Clc-7↑;	Increased osteoclasts; Bone mass loss	(9)
Bone formation	Decreased osteoblast differentiation	Runx2, BMP2/4↓	Bone formation↓; Impaired osteogenesis; Reduced bone mass; Disrupted trabecular structure; Abnormal skeletal development	(8)
		OPG, IGF1↓		(9)
		RANKL/OPG ratio↓		(62)
	Reduced osteogenic potential of BMSCs	NF-κB↑; SOX9↓; BMSCs senescence; PPARγ↑	Osteogenic differentiation↓; Reduced osteoblast number	(48, 65)
	Increased osteoblast senescence	BMP-Smad signaling↓; OCN↓	Bone mass loss; Reduced osteoblast number	(59)
Oxidative stress	ROS↑	Caspase-3↑; Bax/Bcl-2↑; Antioxidant genes sod1↓, cat↓	Osteoblast apoptosis	(8)
		RANKL/OPG ratio↓; Wnt/β-catenin↓; NF-κB/MAPK↑	Enhanced osteoclast activity; Impaired osteogenic differentiation; Bone mineralization defects; Osteoblast apoptosis	(3, 62, 79)
		Mitochondrial fusion/fission imbalance (MFN2↑, FIS1↓)	Impaired differentiation of hBM-MSCs into osteoblasts	(75)
Bone marrow microenvironment	Disrupted hematopoietic homeostasis	Decreased hematopoietic function	Disturbed stem cell homeostasis; Bone marrow microenvironment damage	(35, 38)
Skeletal muscle	Decreased myoblast proliferation; Increased apoptosis; Impaired differentiation	Increased β-galactosidase activity; Upregulation of p16, p21, and senescence-associated secretory phenotype (SASP)expression; Cell cycle arrest	Skeletal muscle senescence; Impaired myogenesis; Reduced muscle mass	(94)
	ROS↑	Mitochondrial dysfunction (Mfn1/2↓, DRP1↑)	Muscle atrophy	(93)
	Inhibited differentiation of primary myoblasts	Increased apoptosis and impaired differentiation of primary myoblasts	Reduced skeletal muscle mass	(92)
	Inhibited angiogenesis	THBS1↑ (angiogenesis inhibition)	Abnormal muscle development	(91)

## 5 Research gaps and future perspectives

MNPs have emerged as a global public health concern. Human exposure to microplastics through ingestion, inhalation, and dermal contact is increasing, posing a potential risk to the general population. Such exposure may occur continuously throughout the human lifespan—from infancy to adulthood. Although the long-term effects of microplastics on human bone and bone marrow have not yet been fully established, experimental studies have shown that even at relatively low concentrations in the microgram range (97), MNPs can accumulate in bone tissue and induce damage to bone marrow cells.

A notable limitation of this review lies in the current gaps and uncertainties in understanding the impact of MNPs on the bone microenvironment and skeletal muscle system. First, the dose–response relationship of long-term human exposure to MPs remains unclear. Most available data are derived from animal models or *in vitro* experiments, with a lack of large-scale, systematic epidemiological and clinical studies in humans. This limitation hampers a comprehensive assessment of the actual health risks posed by MPs. Moreover, the concentrations of MPs used in different studies often exceed levels typically encountered in daily life, as higher doses are frequently employed to predict potential toxic effects. This compromises the clinical translatability of such findings. Additionally, inconsistencies in exposure doses, particle types, experimental models, and endpoint indicators across studies contribute to considerable variability and controversy, thereby limiting the comparability and generalizability of the results.

Second, the underestimation of environmental MNPs exposure remains a major challenge. While humans are chronically exposed to microplastics, current assessments are largely based on dietary intake, often overlooking other critical exposure routes such as inhalation, dermal absorption, and inadvertent ingestion from presumed plastic-free sources. This narrow focus on estimated quantities inevitably results in an underestimation of the total exposure burden. Unlike short-term laboratory studies, real-world human exposure to microplastics is lifelong, beginning in the embryonic stage and extending into old age, raising growing concerns about their cumulative effects. Another often overlooked source of microplastic exposure involves unavoidable contact through medical, dental, and cosmetic procedures. The degradation behavior of microplastics within biological systems significantly influences their toxicity and bioaccumulation. However, the underlying degradation mechanisms and their implications for the selection of bone repair materials remain poorly understood. Degradation products of microplastics may alter the biocompatibility and mechanical properties of such materials, providing important considerations for the design and safety evaluation of biomedical implants.

Currently, research on the detrimental effects of micro- and nanoplastics (MNPs) on the musculoskeletal system has predominantly focused on elucidating toxicological mechanisms, while preventive and therapeutic strategies remain largely underexplored. Preliminary evidence (98) suggests that certain natural antioxidants, such as anthocyanins and quercetin derivatives, may mitigate MNPs-induced oxidative stress and inflammatory responses, indicating their potential in preventing MNPs-related skeletal damage. Moreover, interventions targeting key signaling pathways, including NF-κB, mTOR, and autophagy, have demonstrated restorative effects in experimental models (48, 59), providing a theoretical foundation for the development of targeted pharmacological approaches. Future studies should incorporate exposure models that more closely mimic physiological conditions in humans to systematically evaluate the efficacy and safety of such interventions. In addition, given its low metabolic turnover and high degree of mineralization, bone tissue may serve as a potential reservoir for MNPs accumulation. Recent findings have identified various polymeric microplastics in human bone and bone marrow (39), suggesting that bone may act as a “time capsule,” retaining a long-term record of environmental exposure in individuals or populations. However, the mechanisms governing the deposition, transport, and potential clearance of MNPs within bone tissue remain poorly understood. Whether MNPs can be gradually removed through bone remodeling, or instead persist indefinitely once deposited, has yet to be determined. If bone indeed exhibits high affinity for and retention of MNPs, it may represent a critical site for long-term bioaccumulation, with implications for environmental epidemiology, biomonitoring, and the design of bone repair materials. Therefore, future research should not only address the dose–response relationships, molecular mechanisms, and transgenerational effects of MNPs exposure on the skeletal and muscular systems, but also expand toward the development of individualized and population-level intervention strategies, while recognizing the unique role of bone tissue in toxicity assessment and retrospective exposure analysis.

Against this backdrop, the present review aims to summarize current evidence regarding the exposure and accumulation of MNPs and their effects on the human musculoskeletal system. Future research should focus on developing exposure models that more closely mimic physiological human conditions, and conducting long-term *in vivo* and *in vitro* studies using gradient dosing strategies to elucidate the dose–response relationship and potential transgenerational effects of MNPs. Moreover, there is a critical need to investigate the degradation processes and metabolic dynamics of MNPs with diverse physicochemical properties in bone tissue, to inform the development and application of biomaterials for bone repair. Integrating multi-omics approaches will also be essential to uncover the molecular mechanisms underlying the effects of MNPs on skeletal muscle and the bone microenvironment, ultimately providing a scientific foundation for prevention and therapeutic intervention.
